# Supplementary material for: Direct observation of picosecond melting and disintegration of metallic nanoparticles
Source: Nat Commun. 2019 Jun 3;10:2411. doi: 10.1038/s41467-019-10328-4 (PMC6547703; doi:10.1038/s41467-019-10328-4)
Supplement: Supplementary file 1 — Supplementary Information [file 41467_2019_10328_MOESM1_ESM.pdf]

Supplementary Information:

Direct observation of picosecond melting and disintegration of metallic nanoparticles

Ihm, et al.

This document includes:

Supplementary Figures;

Supplementary Notes;

Supplementary Discussion;

Supplementary References.

Supplementary Figures:

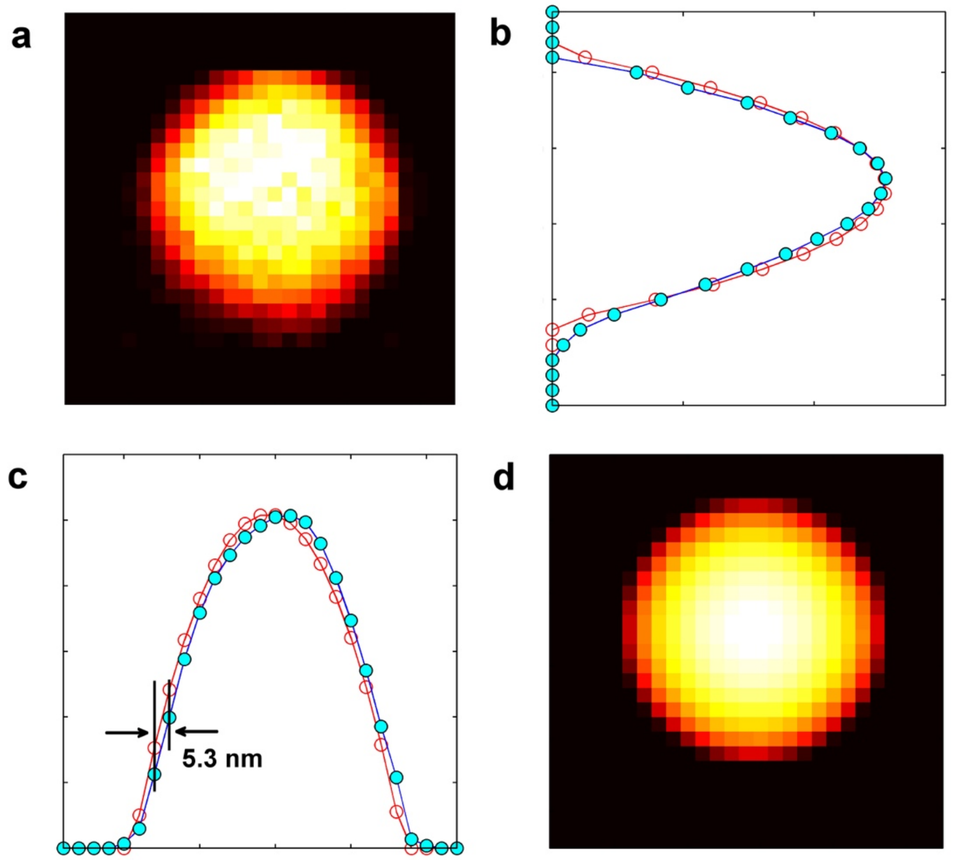

Supplementary Figure 1. Single-shot image of a gold nanosphere. **a-c** Reconstructed image of a gold nanosphere. 1D density distributions are displayed by projecting the obtained image (a) along the horizontal direction in (b) and vertical direction in (**c**) using filled circles. **d** Simulated perfect sphere. Projected densities from simulated sphere in (d) are overlaid in (b) and (c) for a comparison with open circles. Line plots of the projected densities imply that obtained image is close to an ideal perfect sphere at the pixel resolution of 5.3 nm.

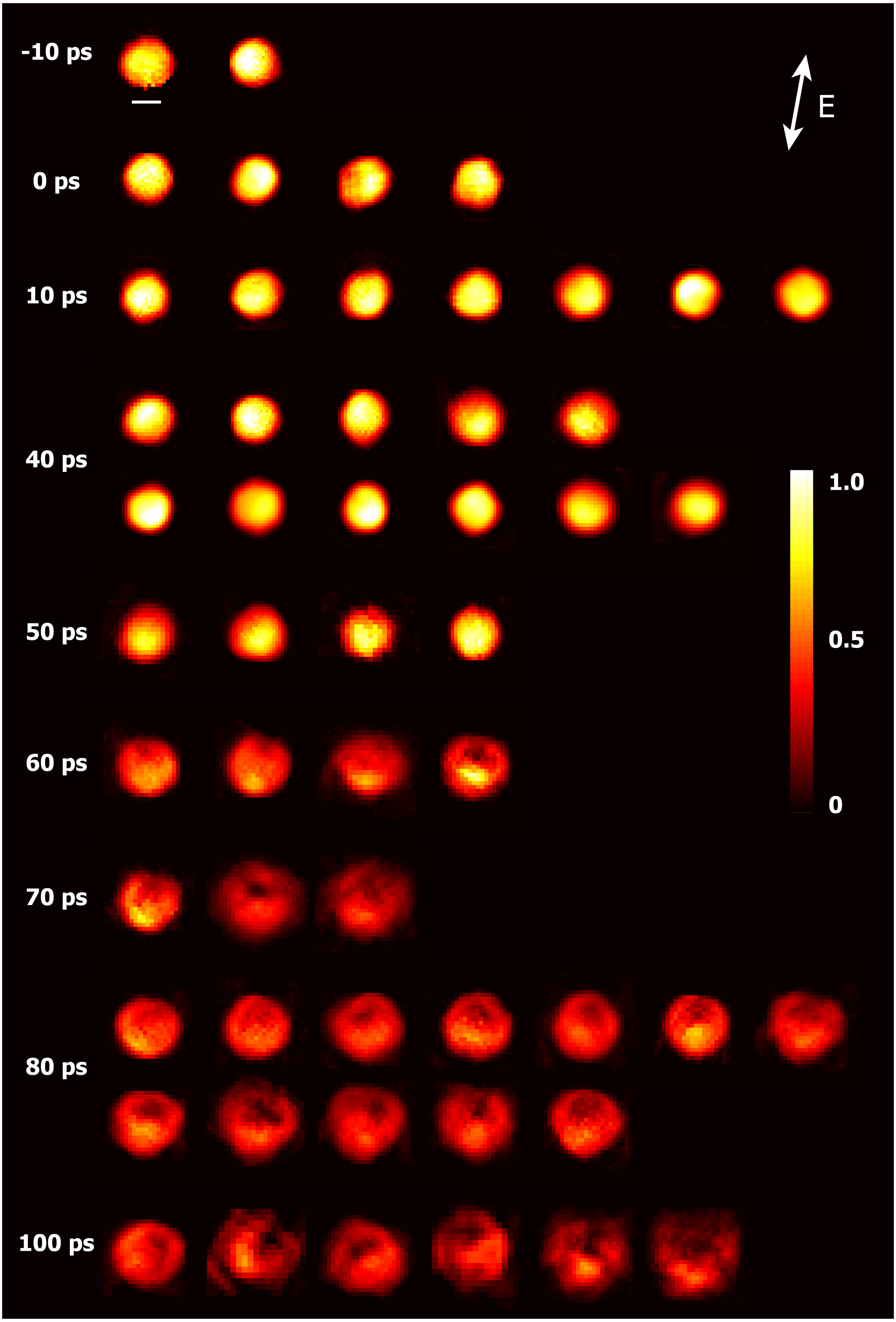

-10 ps
0 ps
10 ps
40 ps
50 ps
60 ps
70 ps
80 ps
100 ps
E
1.0
0.5
0

Supplementary Figure 2. Femtosecond snapshot images of single Au nanospheres on melting and disintegration transitions. Collection of the single shot images independently taken from single Au nanospheres is displayed. All the images are obtained from independent particles exposed to a single laser pulse. Images are sorted for the delay time. A small degree of fluctuation is noted for each single shot image, but overall behavior occurring during the disintegration transformation is consistent with the development of inhomogeneous density variation and void formation with the anisotropy following the fs IR laser polarization direction. All the images with the projected densities are drawn with the same scale for the colormap to have maximum projected density of the intact particle as one with the bright yellow color.

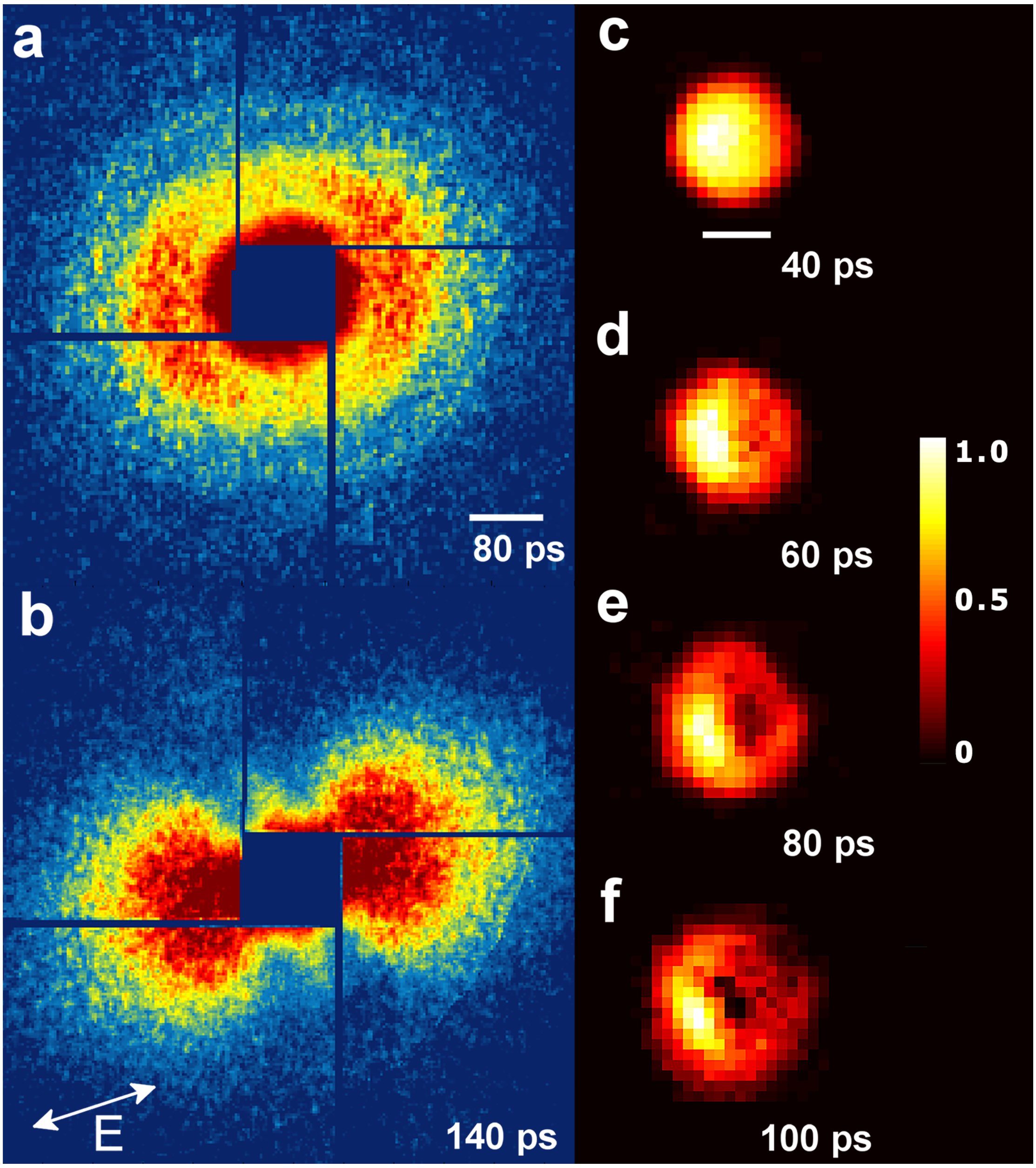

Supplementary Figure 3. Au melting with laser polarization along horizontal direction. **a-b** Single-shot diffraction patterns of single Au particles during melting by an IR pump laser with horizontal polarization. The scale bar in (a) displays 10 1/μm and is the same for (a-b). **c-f** Reconstructed sample images display horizontal shifts of the high-density regions, parallel to the polarization direction. The scale bar in (c) shows 50 nm and is the same for all images in (c-f). Laser polarization direction is shown with an arrow in (b).

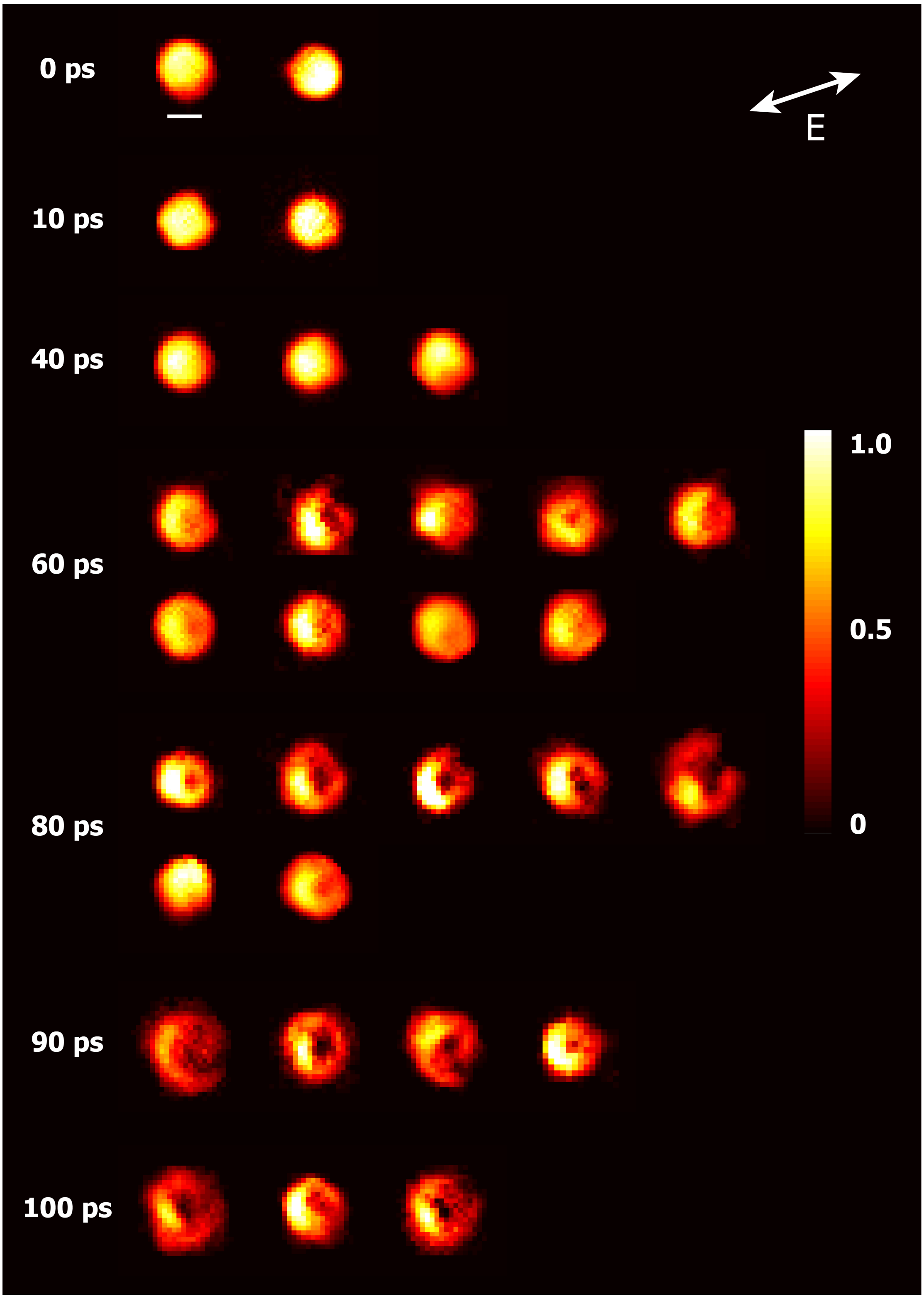
0 ps
10 ps
40 ps
60 ps
80 ps
90 ps
100 ps
E
1.0
0.5
0

Supplementary Figure 4. Femtosecond snapshot images of single Au nanospheres for the horizontal polarization. Collection of the single shot images independently taken from single Au nanospheres is displayed as made for Supplementary Fig. 2 similarly. This time, however, the laser polarization axis is changed to the horizontal direction as indicated in the image. All the images are obtained from independent particles exposed to a single laser pulse. Anisotropic development of inhomogeneous density variation and void follow the newly defined fs IR laser polarization direction. All the images with the projected densities are drawn with the same scale for the colormap to have maximum projected density of the intact particle as one with the bright yellow color.

Supplementary Note 1. Absorption of IR laser pulses by Au nanoparticles

- Taking the refractive index of Au at 800 nm:

$$N_{Au} = \sqrt{\varepsilon} = n + i\kappa = 0.1808 + 5.1173i$$

- The absorption length is 24.9 nm using $\ell_{abs} = \frac{c}{\omega * \kappa}$, and the absorption coefficient is 0.0263 from $A = \frac{4n}{(n+1)^2+\kappa^2}$ (from Landau & Liftshitz)[1]. The absorbed laser fluence is about 14 mJ cm^{-2} from $A * F_{inc} = 0.0263 * \left(0.75 * 17 \frac{\mu J}{\pi * (25\mu m)^2} * 0.82\right) = 0.615 * $ 22.8 mJ cm^{-2}. Average laser intensity (~ 75 %) and geometrical factor (0.82) from the incidence of laser beam with a slanting angle of ~ 35 degree are considered.
- The absorbed energy density is estimated using the absorption length and absorption coefficient above to give ~ 11kJ cm^{-3}.

Supplementary Note 2. Image reconstruction and analysis

The measured data were binned after subtracting the detector noise using data files without x-ray exposure. Image reconstructions were performed after binning the data by 5-by-5 pixels or 7-by-7 pixels numerically. The guided hybrid-input-output algorithm was employed for 16 independent seeds up to the 6th generation. The five best images with lowest errors were averaged to represent the reconstructed image of the data[2]. We have used rectangular supports to avoid any biased reconstruction using circular shaped supports. Radii of the particles at different time delays (Fig. 3b) were obtained by fitting the image boundaries, representing whole sample morphologies, to a circle.

Supplementary Note 3. Atomistic two-temperature molecular dynamic simulations

Molecular dynamics simulations in this work were performed with Au spheres of 10 nm in diameter and the data for 100 nm Au sphere melting were derived by incorporating size effects as described below. A 10 nm Au sphere was placed at the center of a cubic simulation box of 100 nm, which was divided into spherical grids with $N_r = 55$, $N_\theta = 10$, and $N_\varphi = 10$. The sizes of the polar and azimuthal grids were uniform. In order to minimize uneven distribution of grid atom numbers, the spacings for the first ten radial grids containing the initial Au atoms were set to be inversely proportional to the radial distance, beyond which a uniform spacing of 1 nm was used. This gives, for the first ten radial grids, the n-th radial grid size and distance to be $\Delta r(n) = A/n$ and $r(n) = \sum_{i=1}^{n} \Delta r(i) - \Delta r(n)/2$, respectively. The constant A is determined from $\sum_{i=1}^{10} \Delta r(i) = r_0$ with $r_0 = 0.5\ nm$ (the initial Au sphere radius).

A molecular dynamics code[3] was adapted to implement TTMD simulations. TTMD combines molecular dynamics with the two-temperature model that deals with the nonequilibrium states of the electronic and ionic systems at two-temperature level. In two-temperature model, the electronic part is described at the continuum level by the following finite difference (FD) equation, in which each term of the right-hand side represents the electronic heat conduction (first), energy transfer from the lattice (second), and energy absorption from the source (third),

$$C_e \frac{\partial T_e}{\partial t} = \nabla \cdot (K_e \nabla T_e) - G(T_e - T_l) + S(\vec{\boldsymbol{r}}, t) \quad (1)$$

where C_e, K_e are the electron heat capacity and thermal conductivity, G, S the electron-phonon coupling constant and absorbed energy from the source, T_e, T_l the electron and

lattice temperatures. The electron temperature-dependent C_e and G of gold were obtained from Lin et al.[4]. The electron thermal conductivity of gold shown in equation (Supplementary Equation 2) was obtained from Ivanov and Zhigilei[5].

$$K_e(T_e) = \frac{1}{3} v_F^2 \frac{\gamma T_e}{A T_e^2 + B T_l} \tag{2}$$

where $A = 1.2 \times 10^7 K^{-2} s^{-1}, B = 1.23 \times 10^{11} K^{-1} s^{-1}, \gamma = 71\, J\, m^{-3} K^{-2}, v_F = 4.0 \times 10^5\, ms^{-1}$. The absorbed energy by the grid (*i* ,*j*, *k*) at *t* is the following:

$$S(\boldsymbol{r}, t) = f_a \frac{F_{abs}}{\tau l_p} \sqrt{\frac{4 \ln 2}{\pi}} \exp\left[\frac{r - r_0}{l_p}\right] \exp\left[-\frac{(t - t_0)^2}{2\sigma^2}\right] \tag{3}$$

where $F = 14\, mJ\, cm^{-2}, \tau = 50\, fs, l_p = 2.49\, nm, \sigma = 21\, fs$ are absorbed laser fluence, pulse width, absorption length, and the standard deviation of the Gaussian pulse profile, respectively, which are consistent with the experimental conditions, with the absorption length scaled by the sphere size. The prefactor, f_a , is the anisotropy factor of the energy absorption, which is one for an isotropic energy absorption. The following anisotropy factor reflects the energy absorption through anisotropic field enhancements due to Mie scattering and propagating plasma waves[6].

$$f_a(\boldsymbol{r}, t) = \begin{cases} \alpha_1 A cos\theta \exp\left(\frac{r - r_0}{r_0}\right) \left|\cos\left(\frac{t}{T}\right)\right| & \left(0 \le \theta \le \frac{\pi}{2}\right) \\ \alpha_2 A cos(\pi - \theta) \exp\left(\frac{r - r_0}{r_0}\right) \left|\sin\left(\frac{t}{T}\right)\right| & \left(\frac{\pi}{2} < \theta \le \pi\right) \end{cases} \tag{4}$$

with $A = 8.3, \alpha_1 = 1.8,\ \alpha_2 = 0.38,\ T = 20\, fs$, which are the normalization factor, enhancement factors for each hemi-sphere, and the oscillation period. The electron

temperature evolution is determined for each grid by solving the FD equation (Supplementary Equation 1), whose time step is smaller than the MD time step[5].

At each MD step, electron temperatures are redefined to satisfy the total energy conservation when atoms move between grids at subsequent MD steps; $T_e^n(i,j,k) = \frac{1}{N(i,j,k)}\sum_{q=1}^{N(i,j,k)} T_e^{n-1}(i'_q, j'_q, k'_q)$. Here *n, q,* and *N(i,j,k)* are MD step, atom index, the number of atoms of grid *(i, j, k)*, and (i'_q, j'_q, k'_q) the grid indices of atom q at (n-1)-th MD step. The total energy transfer from the electronic to ionic system of grid *(i, j, k)* at each MD step is obtained with summation of the second term in the right-hand side of the equation (Supplementary Equation 1) over FD time step:

$$\Delta E(i,j,k) = \sum_{\alpha=1}^{N_{FD}} G^{\alpha}(i,j,k)\,(T_e^{\alpha}(i,j,k) - T_l^{\alpha}(i,j,k))V(i,j,k)(\frac{\rho(i,j,k)}{\rho_0})\Delta t_{FD} \quad (5)$$

where $\alpha, \Delta t_{FD}, N_{FD}, V(i,j,k)$ are the FD step index, time step, the number of FD steps in each MD step, and the volume of grid (*i, j, k*). This energy transfer into the ionic system is added to the lattice in the next MD step by rescaling the atomic velocities: $\vec{v}(\vec{\boldsymbol{r}}, t + \Delta t_{MD}) = \gamma(i,j,k,t)\vec{v}(\vec{\boldsymbol{r}}, t)$, with the velocity rescaling constant of grid (*i, j, k)* obtained from the initial kinetic energy and the energy transfer to lattice: $\gamma(i,j,k,t) = \sqrt{(K(i,j,k) + \Delta E(i,j,k))/K(i,j,k)}$.

The speed of heterogeneous melting is limited by the speed of sound, thereby the melting progress is expected to correlate with sphere size. Our analysis with 10, 15, 20 Å Au spheres with isotropic energy absorption gives a linear relationship between central void formation time and sphere size: $t(D) = -3.5 + 0.835D$ with D the nanosphere size. From this relation, the temporal evolution of melting for 10 nm sphere

(t^*) was converted to that of 100 nm sphere (t): $t = 16\, t^*$. All the comparisons with the experimental data are based on t.

Supplementary Note 4. Order parameter calculation

The crystalline and disordered phases are determined based on the order parameters calculated following the method of Morris and Song[3].

$$\psi = \left|\frac{1}{N_q}\frac{1}{N_n}\sum_{\boldsymbol{q}}\sum_{\boldsymbol{r}} \exp\left(i\boldsymbol{q}\cdot\boldsymbol{r}\right)\right| \tag{6}$$

where **q** represents N_q wave vectors satisfying $e^{i\boldsymbol{q}\cdot\boldsymbol{\xi}} = 1$ for the vectors $\boldsymbol{\xi}$ connecting near neighbors in the perfect fcc lattice. ***r*** represents the vectors connecting the atom and surrounding atoms at distances between nearest and next nearest neighbor in perfect fcc lattice. N_q=6 and N_n=18 were used. The cutoff of $\psi = 0.04$ was used for determination of the crystalline and liquid phases[5].

Supplementary Discussion. TTMD results and interpretation

Projected density maps of ions obtained from the TTMD simulations show good agreement with experimental results (Figs. 3a and 4a). Initially, the ion density became diluted indicating the melting from the surface or more dominantly from the pole region of a hemisphere parallel to the laser polarization direction (Fig. 4a). Next, this melting front propagated toward the center of the nanoparticle and developed an off-centered void (~ 80 and 100 ps), which was followed by a complete destruction of the nanoparticles. Detailed development process of the melting at the atomic scale can be characterized by the disorder of Au ions from the perfect crystal lattice of face centered cubic (fcc) symmetry. We calculated the order parameter for each individual ion using

the displaced positions of all the Au ions obtained from the TTMD simulations (Fig. 4b). The order parameter is defined as the normalized phase sum of atomic position vectors in relation to the perfect lattice (Supplementary Note 4)[3]. A perfect crystal has an order parameter of one and any value smaller than 0.04 is defined as the loss of crystalline order. The order parameter map shows that the fs pump laser initiated asymmetric disordering from a pole region at the surface as early as ~10 ps (Fig .4b and Supplementary Movie 3), which was barely notable in the projected density maps obtained from experiments (Fig. 3) and MD simulations (Fig. 4a). The melting front initiated from the north-pole reached the center in ~20 ps, and all the ions became disordered in 40 ps, after which the void developed and propagated toward the center (Fig. 4).

Supplementary References

1 Landau, L. D., Lifshitz, E. M. & Pitaevskii, J. P. *Electrodynamics of Continuous Media*. (Pergamon Press, 1984).

2 Nam, D. *et al.* Imaging Fully Hydrated Whole Cells by Coherent X-Ray Diffraction Microscopy. *Phys. Rev. Lett.* **110**, 098103 (2013).

3 Morris, J. R. & Song, X. The melting lines of model systems calculated from coexistence simulations. *J. Chem. Phys.* **116**, 9352-9358 (2002).

4 Lin, Z., Zhigilei, L. V. & Celli, V. Electron-phonon coupling and electron heat capacity of metals under conditions of strong electron-phonon nonequilibrium. *Phys. Rev. B* **77**, 075133, doi:10.1103/PhysRevB.77.075133 (2008).

5 Ivanov, D. S. & Zhigilei, L. V. Combined atomistic-continuum modeling of short-pulse laser melting and disintegration of metal films. *Phys. Rev. B* **68**, 064114 (2003).

6 Varin, C., Peltz, C., Brabec, T. & Fennel, T. Attosecond Plasma Wave Dynamics in Laser-Driven Cluster Nanoplasmas. *Phys. Rev. Lett.* **108**, 175007 (2012).
