# Supplementary material for: Direct observation of picosecond melting and disintegration of metallic nanoparticles
Source: Nat Commun. 2019 Jun 3;10:2411. doi: 10.1038/s41467-019-10328-4 (PMC6547703; doi:10.1038/s41467-019-10328-4)
Supplement: Supplementary file 3 — Description of Additional Supplementary Files [file 41467_2019_10328_MOESM3_ESM.pdf]

Description of Additional Supplementary Files

Supplementary Movie 1

- Filename: Ihm SM1 Au melting.mov
- Title: Nanoscale imaging of picosecond melting and disintegration in irreversible transition.
- Short description: Nanoscale images obtained from XFEL single-pulse pump-probe imaging experiments show the detailed progress of ultrafast melting and disintegration.

Supplementary Movie 2

- Filename: Ihm SM2 MD melting.mpg
- Title: Two-temperature molecular dynamics simulations of the Au melting
- Short description: The atomistic two-temperature molecular dynamics simulations show the dynamics of Au nanoparticle on melting after anisotropic energy absorption from single fs IR laser pulse.

Supplementary Movie 3

- Filename: Ihm SM3 MD order parameters.mpg
- Title: The picosecond evolution of atomic disorder indicated by the order parameter
- Short description: The order parameter for each individual Au atom in the nanoparticle is calculated from the TTMD simulations. Atoms with a perfect crystalline order are colored in red, and disordered atoms are colored in blue.
